# Rate-Dependent Modeling of Piezoelectric Actuators for Nano Manipulation Based on Fractional Hammerstein Model

**DOI:** 10.3390/mi13010042

**Published:** 2021-12-28

**Authors:** Liu Yang, Zhongyang Zhao, Yi Zhang, Dongjie Li

**Affiliations:** 1 School of Automation, Harbin University of Science and Technology, Harbin 150040, China; ZYZhao15245690033@163.com (Z.Z.); ztianyi1997@gmail.com (Y.Z.); dongjieli2013@163.com (D.L.); 2 Heilongjiang Provincial Key Laboratory of Complex Intelligent System and Integration, Harbin University of Science and Technology, Harbin 150040, China

**Keywords:** hammerstein model, bouc-wen model, fractional model, rate correlation, artificial bee colony algorithm

## Abstract

Piezoelectric actuators (PEAs), as a smart material with excellent characteristics, are increasingly used in high-precision and high-speed nano-positioning systems. Different from the usual positioning control or fixed frequency tracking control, the more accurate rate-dependent PEA nonlinear model is needed in random signal dynamic tracking control systems such as active vibration control. In response to this problem, this paper proposes a Hammerstein model based on fractional order rate correlation. The improved Bouc-Wen model is used to describe the asymmetric hysteresis characteristics of PEA, and the fractional order model is used to describe the dynamic characteristics of PEA. The nonlinear rate-dependent hysteresis model can be used to accurately describe the dynamic characteristics of PEA. Compared with the integer order model or linear autoregressive model to describe the dynamic characteristics of the PEA Hammerstein model, the modeling accuracy is higher. Moreover, an artificial bee colony algorithm (DE-ABC) based on differential evolution was proposed to identify model parameters. By adding the mutation strategy and chaos search of the genetic algorithm into the previous ABC, the convergence speed of the algorithm is faster and the identification accuracy is higher, and the simultaneous identification of order and coefficient of the fractional model is realized. Finally, by comparing the simulation and experimental data of multiple sets of sinusoidal excitation with different frequencies, the effectiveness of the proposed modeling method and the accuracy and rapidity of the identification algorithm are verified. The results show that, in the wide frequency range of 1–100 Hz, the proposed method can obtain more accurate rate-correlation models than the Bouc-Wen model, the Hammerstein model based on integer order or the linear autoregressive model to describe dynamic characteristics. The maximum error (Max error) is 0.0915 μm, and the maximum mean square error (RMSE) is 0.0244.

## 1. Introduction

PEA is a smart material with excellent performance. It has outstanding advantages such as large force, high rigidity, high control accuracy, low power consumption, and fast response speed. Therefore, it has been widely used, such as in micro-manipulation [1], a micro-mechanical arm [2], active optical components [3], active vibration control [4], biomedical engineering [5], etc. In the application of PEA for active vibration control, it is mainly aimed at the low-frequency vibration with a frequency below 100 Hz, at which time the traditional passive vibration isolation method is difficult to work [6]. PEA is adopted to produce motion with the same size and opposite direction as the vibration wave, thus weakening the vibration amplitude. In this process, PEA is required to accurately track the reference signal with random and continuous changes in amplitude and frequency. Therefore, the modeling accuracy of its nonlinear characteristics such as hysteresis and creep, as well as the variation rule of these nonlinear characteristics following the motion frequency, will significantly affect the performance of the control method based on inverse model compensation. In the existing literature, although some research results are given for the modeling and control methods of rate dependent nonlinear characteristics of PEA at different frequency points. The existing modeling methods still have great shortcomings for the description of nonlinear characteristics of continuous random variation in tracking frequency. Therefore, it is of important research value to carry out more accurate modeling methods for the rate-dependent nonlinear characteristics of PEA [7].

Scholars at home and abroad have done a lot of research on the hysteresis modeling of piezoelectric actuators. At present, based on the classic hysteresis model and the development of computational intelligence, the modeling methods of hysteresis characteristics can generally be divided into three types. (1) Proceeding from the physical principles and based on the physical mechanism, give obvious physical models, such as the Maxwell model [8], Duhem model [9], Jiles-Atherton model [10], Stoner Wohlfarth model [11], and finite element model [12], etc. For example, in literature [13,14], the authors obtained the finite element model with specific physical mechanism by analyzing the piezoelectric effect and constitutive model of PEA, so the description of hysteresis nonlinearity is more accurate. However, for the hysteresis nonlinear system of different objects, a model corresponds to a physical mechanism. So, it can usually only describe a specific object, and the generality is not strong, and the parameters of the model depend on the physical parameters of the object, which is not easy to identify online. (2) Consider the establishment of the model from the actual input and output, without considering the actual physical meaning of the phenomenological model, such as the Preisach model [15], Pradtl-Ishlinskii model [16], Krasnoselskii-Pokrovskii model, [17] and so on. The phenomenological model relies on the input and output relationship of the system to perform hysteresis modeling, and describes the entire system as a black box. Among them, the core idea of the Pradtl-Ishlinskii model in literature [15] is the weighted superposition of PI operators. The literature [17] points out that the KP operator is an extension of the PI operator, and the Krasnoselskii-Pokrovskii model is also a weighted superposition of the KP operator. This type of model is relatively simple, but it requires a large number of experiments as a basis, and it is difficult to adjust the parameters online. And it is difficult to apply. (3) Models that describe input and output based on intelligent computing, such as the artificial neural network model, support the vector machine model, etc. The intelligent model uses the good approximation performance of intelligent algorithms to model some nonlinear systems with high modeling accuracy. But the neural network modeling method [18] does not have a general standard to determine the optimal structure of the neural network, such as the number of hidden layers, the number of neurons in each layer, etc., and the neural network can easily fall into a local optimum. The hysteresis modeling method of support vector machine [19], which has the advantages of global optimization and versatility. But this method is limited to offline identification.

The Hammerstein model, as a class of nonlinear dynamic models composed of nonlinear static modules and linear dynamic modules, has been proven to describe a large class of nonlinear systems [20,21]. Many studies realize the static inverse compensation or dynamic inverse compensation of hysteresis nonlinearity based on this method. However, when identifying the parameters of the model, different identification algorithms will also have an impact on the accuracy of the model. At present, most Hammerstein models adopt a combination of traditional hysteresis models and integer-order or linear autoregressive models to model PEA, and traditional identification algorithms are usually used for model parameter identification. Zhou, M. and Wang, J. applied a recursive least squares algorithm and gradient correction algorithm in the identification of Duhem model [22]. Yu, S., Feng, Y. and Yang, X. used particle swarm optimization (PSO) in the identification of Bouc-Wen model [23]. Wang, Z. and Zhen, Z. et al. used PSO [24] in the Hammerstein model composed of Bouc-Wen model and linear autoregressive model (ARX). Wang, G. and Chen, G. et al. apply the differential evolution algorithm (DE) in the identification of the Bouc-Wen model [25]. Although the single identification algorithm mentioned above can identify the parameters of the model, its premature phenomenon, which fast convergence to the local optimal solution rather than the global optimal solution, swing near the optimal solution when approaching the optimal solution, and slow convergence speed, will affect the identification accuracy of the model, and then the accurate model cannot be obtained.

In response to the above problems, the main research content and contributions of this article can be summarized as follows:

1. In terms of PEA rate-dependent modeling, this paper proposes a modeling method based on the improved Bouc-Wen model and fractional-order model consisting of a separate Hammerstein model, which achieves a more accurate description of the dynamic characteristics of PEA rate-dependent hysteresis. At present, most Hammerstein models adopt a combination of traditional hysteresis models and integer-order or linear autoregressive models to model PEA. When using integer-order or linear autoregressive models to describe the dynamic characteristics of PEA, the accuracy is far inferior to that of fractional-order models. Compared with the integer-order or linear autoregressive models, it is more in line with engineering reality and can contain richer amplitude-frequency information.
2. In terms of model parameter identification, this paper proposes an artificial bee colony algorithm based on differential evolution (DE-ABC), and uses it for the first time for the parameter identification of the fractional Hammerstein model. By adding the mutation strategy and chaos search for genetic algorithm into the previous ABC, the convergence speed of the algorithm is faster and the identification accuracy is higher, and the simultaneous identification of order and coefficient of fractional model is realized.
3. Through experimental data collection and analysis, the proposed model has a good frequency generalization ability within 1–100 Hz, and can better reflect the true characteristics of piezoelectric ceramic actuators than the traditional static hysteresis model.

The paper’s structure is arranged as follows. Section 2 depicts the model structure, consisting of an improved Bouc-Wen model, a fractional-order model, and a rate-dependent Hammerstein model. Section 3 proposes to use the artificial bee colony algorithm based on differential evolution for the identification of the improved Bouc-Wen static hysteresis nonlinear model and the fractional dynamic model. Finally, Section 4 introduces the experimental design, compares the identification results with different algorithms, and validates the Hammerstein model and piezoelectric actuator. The staple conclusions are summed up in Section 5.

## 2. Fractional Hammerstein Model of PEA

**Figure 1:**
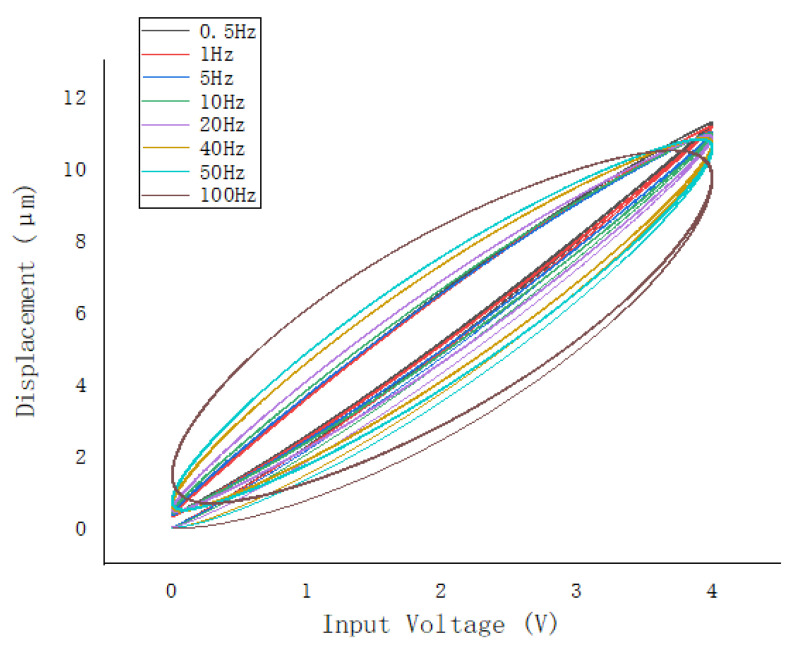
Rate-dependent hysteresis characteristics of piezoelectric actuator (PEA).

The rate-dependent hysteresis system refers to a system whose output is related to the current and previous inputted signals and their frequency. As shown in Figure 1, PEA have rate-dependent hysteresis nonlinearity, that is, when the piezoelectric actuator changes greatly in the frequency of the input voltage, the relationship between its input and output changes greatly.

**Figure 2:**
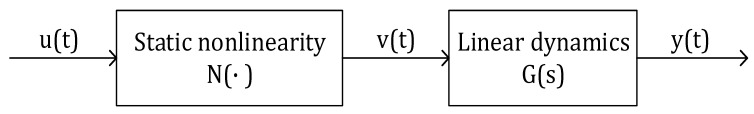
The structure of the classic Hammerstein model.

At present, there is no unified mathematical model for nonlinear systems. As a nonlinear model based on module connection, the Hammerstein model can more precisely depict the rate-dependent characteristics of PEA. The classic Hammerstein model [24,26] contains static nonlinearity and linear dynamic model [27,28]. Its structure diagram is shown in Figure 2.

**Figure 3:**
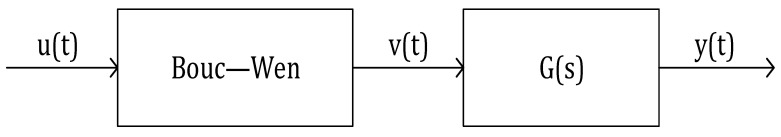
Hammerstein model structure of the PEA.

Experiments show that when the frequency of the drive signal is low, the hysteresis curve of the PEA shows rate-independence; when the frequency is high, the hysteresis curve shows rate-dependence. So as to bewrite the rate-dependent hysteresis nonlinearity of PEA, this paper proposes a rate-dependent hysteresis nonlinear model based on the Bouc-Wen model and fractional-order model. Among them, the linear dynamic part of the Hammerstein model is described by the fractional-order model, and the static nonlinear part is described by the Bouc-Wen model. The structure is shown in Figure 3.

### 2.1. The Improved Bouc-Wen Model

The classic Bouc-Wen model was introduced by Bouc [29], and Wen [30] extended it. The Bouc-Wen model contains fewer parameters and can be recognized by less data than other models depicting hysteresis characteristics. But in reality, the PEA input has an inherently multi-value memory dependent hysteresis loop asymmetry. Therefore, the improved Bouc-Wen model is adopted in this paper. By defining l[u(t)] as a generalized input function, that is, the function of input voltage u(t), it is used to describe the asymmetric property of PEA input and output about origin asymmetry. The improved Bouc-Wen model expression is:

(1) $$\begin{array}{l} x\left(t\right)=l\left[u\left(t\right)\right]-z\left(t\right)=\left[xu^{2}\left(t\right)+yu\left(t\right)\right]-z\left(t\right)\\ \dot{z}\left(t\right)=\alpha \dot{u}\left(t\right)-\beta \left|\dot{u}\left(t\right)\right|z\left(t\right){\left|z\left(t\right)\right|}^{n-1}-\gamma \dot{u}\left(t\right){\left|z\left(t\right)\right|}^{n} \end{array}$$

In the formula, x(t) is the output displacement; l[u(t)] is a nonsingular input function; z(t) is the hysteresis displacement component; $\dot{z}\left(t\right)$ is the derivative of z(t) with respect to time; u(t) represents the input voltage; $\dot{u}\left(t\right)$ is the derivative of u(t) with respect to time; x and y control the asymmetry of input signals; α controls the size of the hysteresis loop; β and γ respectively control the shape of the hysteresis loop; n represents the smoothness of the transition from the elastic part to the sculpted part. By appropriately selecting model parameters, it can express various hysteresis in various shapes.

However, The improved Bouc-Wen model still does not have the rate-independent features. Considering these limitations, modelling errors cannot be ignored in practical applications [31].

### 2.2. Fractional Dynamic Model

Most Hammerstein models use integer-order calculus models or linear autoregressive model (ARX) models to describe the dynamic characteristics of PEA, which often overlook some real phenomena and properties with fractional-order characteristics. While using the ARX model to describe dynamic characteristics, the output at the current moment is not only determined by the input at the current moment, but also by the input and output at all previous moments. The fractional-order model can be a good substitute for the ARX model due to its long memory characteristics. When using an integer-order model to describe the dynamic characteristics, it is necessary to analyze the dynamic characteristics of the PEA and to estimate the redundant parameters that affect the robustness of the control application. However, the fractional-order system is considered to have improved robustness in the control design. Moreover, due to the mathematical characteristics of fractional globality and long memory, it not only has the advantages of the ARX model but also can use fewer parameters to model complex systems. It has been widely used in physics and control [32,33]. The fractional Hammerstein model avoids complicated internal mechanism analysis on the basis of obtaining good modelling accuracy.

Fractional calculus is the generalization of integer order integration and differentiation to all real numbers. The basic operator ${\;}_{t0}D_{t}^{\alpha }$ is defined as [34]:

(2) $${\;}_{t0}D_{t}^{\alpha }=\{\begin{array}{cc} \frac{d^{\alpha }}{{dt}^{\alpha }}, & Re(\alpha )>0\\ 1, & Re(\alpha )=0\\ \int_{0}{\left(d\tau \right)}^{-\alpha }, & Re(\alpha )>0 \end{array}$$

Among them, $t_{0}$ is the lower limit and t is the upper limit of the integral, α is the fractional- order, which can be a complex number, and Re(α) is the real part of α.

There are many definitions of fractional calculus, among which the most commonly used are the Grunwald–Letnikov (G-L) definition. This paper uses the approximate calculation defined by G-L to carry out the numerical simulation of fractional operators. The definition of G-L is given as [34]:

(3) $${\;}_{t0}D_{t}^{\alpha }f\left(t\right)=\underset{h\to 0}{lim}h^{-\alpha }\times \overset{\left[\left(t-t_{0}\right)/h\right]}{\underset{j=0}{\sum }}{\left(-1\right)}^{j}\frac{\Gamma \left(\alpha +1\right)}{\Gamma \left(\alpha -j+1\right)\Gamma \left(j+1\right)}f\left(t-jh\right)$$

Fractional systems can be represented by fractional linear differential equations, which have the form:

(4) $$a_{0}D^{\alpha_{0}}x\left(t\right)+\cdots +a_{n}D^{\alpha_{n}}x\left(t\right)=b_{0}D^{\beta_{0}}u\left(t\right)+\cdots +b_{n}D^{\beta_{n}}u\left(t\right)$$

Among them, u(t) and x(t) are system input and system output. $D^{\alpha }x\left(t\right)$ is the α-th time derivative of x(t).

If the initial condition is zero, the Laplace transform of $D^{\alpha }x\left(t\right)$ is:

(5) $$L\left\{D^{\alpha }x\left(t\right)\right\}=s^{\alpha }X$$

Take the Laplace to transform on both ends of the above Equation (4). The fractional-order linear time-invariant system can be rewritten as the below transfer function form:

(6) $$G\left(s\right)=\frac{X\left(s\right)}{U\left(s\right)}=\frac{b_{0}s^{\beta_{0}}+\cdots +b_{m}s^{\beta_{m}}}{a_{0}s^{\alpha_{0}}+\cdots +a_{n}s^{\alpha_{n}}}\;\;$$

where $\alpha_{0}<\alpha_{1}<\cdots <\alpha_{n}$, and $\beta_{0}<\beta_{1}<\cdots <\beta_{m}$.

## 3. Artificial Bee Colony Algorithm Based on Differential Evolution (DE-ABC)

**Figure 4:**
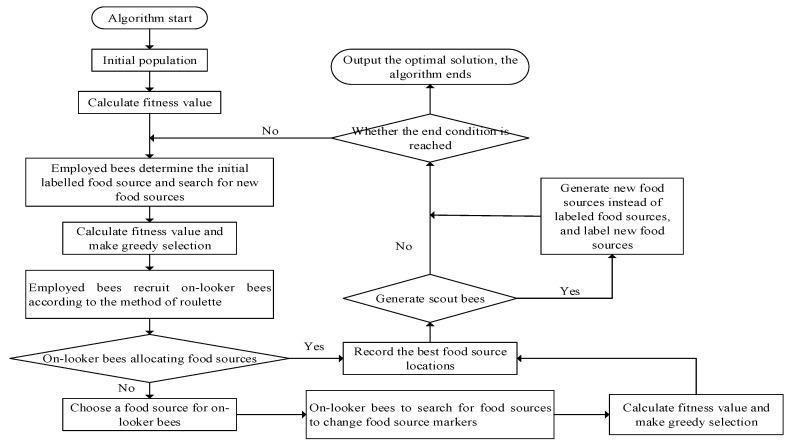
Flow chart of DE-ABC.

Due to the complexity of the fractional systems, the dynamic model using fractional-order description is not easy to estimate. In addition, due to the existence of multiple variables in the problem, there are multiple local search optimal solutions in the objective function, which is effortless to fall into the local optimal solution, and the amount of calculation is large. Practice results show that the traditional differential evolution algorithm or artificial bee colony algorithm is prone to problems such as premature maturity or slow convergence rate [35]. In addition, as the parameters of the model that needs to be identified increases, the algorithm will deteriorate the search space, and the modal error will increase with the increase in complexity. Therefore, it is difficult to efficiently and accurately search for the global optimal solution using traditional general methods. Thus, to solve this problem, this paper uses an effective artificial bee colony algorithm based on the differential evolution strategy (DE-ABC).The flowchart of DE-ABC is shown in Figure 4.

The calculation process of ABC mainly includes three stages: employed bees, on-looker bees and scout bees. When using ABC to identify model parameters, each food source represents a feasible solution to the model parameters, the amount of nectar(fitness) represents the quality of the solution, and the number of solutions is equal to the number of leading bees. In this paper, the root mean square error (RMSE) is introduced as the fitness function of DE-ABC to reflect the modeling error, as shown in Formula (7):

(7) $$J(x_{i})=\sqrt{\frac{1}{N}\sum_{i=1}^{N}{\left(y_{\exp}(i)-y(i)\right)}^{2}}$$

Among them *y*_exp_ is the sampled value of the experimental data, is the sampled value of the model data, *N* is the number of sampling points, each solution $x_{i}\;$(*I* = 1, 2, …) is represented by a D-dimensional vector $x_{i}={\left(x_{i1},x_{i2},\ldots ,x_{iD}\right)}^{T}$, where D is the number of model parameters to be identified.

The specific process of DE-ABC identification is as follows:

1. Obtain the input and output data of the experiment.
2. Set the initial conditions and the number of parameters.
3. Parameter identification based on DE-ABC.
4. Verification. If the fitness does not meet the requirements, return to step 2 to continue identification.

DE-ABC is to introduce the mutation strategy of the differential evolution algorithm into ABC. In the search of the lead bee, the search of the lead bee is carried out according to the Formula (8).

(8) $$x_{ij}^{\prime }=x_{best,j}+F_{i}\times \left(x_{ij}-x_{kj}\right)\;$$

Here, $x_{best,j}$ is the best individual of the previous generation, j∈{1,2,…,D}, k∈{1,2,…,N}, j and k are selected randomly, but *k ≠ j*, $F_{i}$ is no longer a constant used in traditional ABC, but as shown in the Formula (9), it is an adaptive dynamic adjustment variable. Among them, Iter is the maximum number of iterations, and iter is the current number of iterations.

(9) $$F_{i}=1-\frac{iter}{Iter}\;$$

In the early stage of algorithm evolution, iter is smaller, $F_{i}$ is larger, and the algorithm mutation intensity is larger, so that it can evolve to the optimal value more effectively and quickly. As the evolution continues, to the later stage of the algorithm evolution, iter becomes larger and $F_{i}$ becomes smaller. As the individual evolves toward the optimal value, the function can quickly and stably converge to the optimal value.

In traditional ABC, if a certain solution $x_{i}$ does not improve after L cycles, this solution will be abandoned by the employed bees, and the employed bees will become scout bees and randomly generate a new solution instead. In the DE-ABC used in this article, chaotic search is introduced into the identification algorithm.

When a solution is still not improved after L cycles, it may fall into a local optimum, and the scout bees will perform a chaotic search to jump out of the local optimum. The chaotic search here uses the chaotic sequence generated by the Logistic chaotic map instead of the random number in the traditional ABC formula.

The logistic chaotic mapping equation is as follows:

(10) $$C_{n+1}=4C_{n}\left(1-C_{n}\right)$$

Among them, $0<C_{n}<1$. Suppose the solution of search stagnation is $x_{i}=\left(x_{i1},x_{i2},\ldots ,x_{iD}\right)$, the main steps of chaos search of the scout bees are as follows:

1. The initial value of the chaotic sequence generated according to Formula (11);
  (11) $$C_{ij}^{0}=\frac{x_{ij}-x_{min,j}}{x_{max,j}-x_{min,j}}$$
2. Generate chaotic sequence according to Formula (10);
3. Generate a new solution according to Formula (12), calculate its fitness value, compare it with the original solution, and keep the best solution;
  (12) $$x_{ij}^{’}=x_{i,j}+c_{ij}\times \left(x_{max,j}-x_{min,j}\right)$$
4. If the maximum number of chaotic iterations is reached, the search ends, otherwise go to step 2.

## 4. Model Verification

The below paper is about that the Hammerstein model is applied to model the rate-dependent hysteresis characteristics of the PEA under input signals of different frequencies.

### 4.1. Experimental Setup

**Figure 5:**
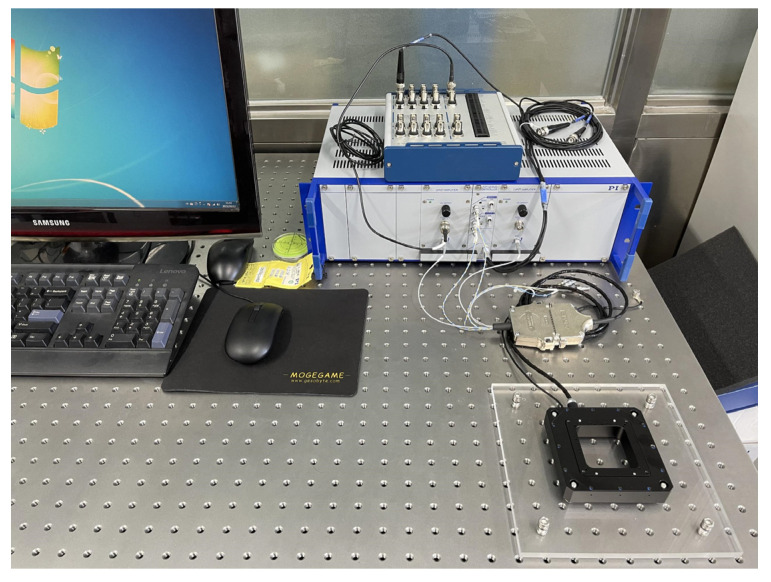
Experimental equipment.

Experimental equipment for the data acquisition experiment of PEA is shown in Figure 5. This equipment consists of a computer, a data acquisition card, a drive power supply, a piezoelectric micro-positioning platform, a piezoelectric amplification module, a piezoelectric control module, and a displacement sensor. The data acquisition card is the USB-6346(BNC) produced by NI. The piezoelectric micro-positioning platform is P733.2DD [36] produced by the PI company. The platform comes with a displacement sensor, the piezoelectric amplifier module E-505.00 produced by PI company, and the piezoelectric control module is an E-509.C2A produced by PI company. In order to obtain the experimental data, the voltage signal generated by the computer MATLAB software is outputted through USB-6346(BNC) and transmitted to the E-509.C2A piezoelectric control module. The control voltage generated is amplified by the E-505.00 amplification module as the driving voltage of the piezoelectric micro-positioning platform. At that moment, the corresponding output displacement of the platform is gauged by the displacement sensor of the P733.2DD platform and then transmitted back to Matlab via USB-6346(BNC) for storage.

### 4.2. Comparison of Model Identification Effects

While the input signal frequency is lower than 5 Hz, the experimental results show that the hysteresis loop of the PEA hardly changes. Therefore, the static Bouc-Wen model is established by taking the input and output data of the PEA at an input frequency of 1 Hz, which reflects the static hysteresis characteristics of the piezoelectric actuator. In order to compare the effects of different identification algorithms, the traditional differential evolution algorithm (DE), ABC and DE-ABC are employed to discern the parameters of the improved Bouc-Wen model.

**Figure 6:**
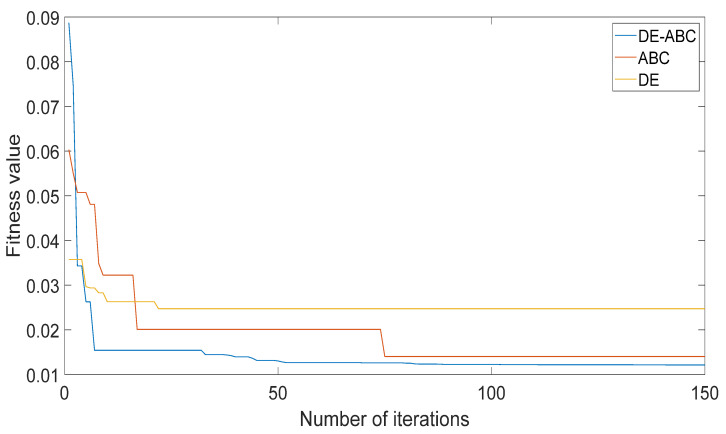
Algorithm comparison.

In order to realize the improved Bouc-Wen model identification process, the initial values of relevant parameters must firstly be determined. Through trial and error, the upper and lower circumscriptions of the explore scope are set to MinX = [2 0 1 1 0], MaxX = [1 −2 0 0 −1], and the number of iterations is set to 150 generations. Compared with the experimental data, the DE-ABC is superior to the DE and the ABC in terms of convergence rate and identification accuracy. The effects of the above three algorithms are shown in Figure 6 and Table 1.

### 4.3. Fractional Hammerstein Model

**Figure 7:**
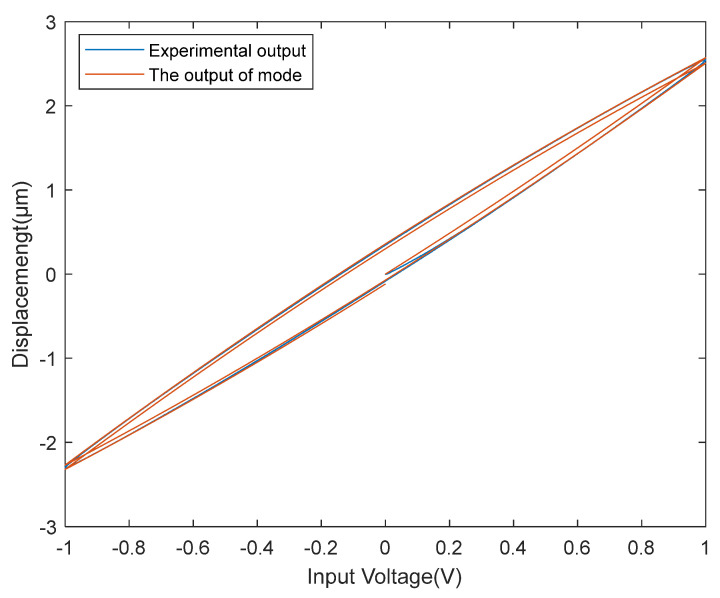
The input and output curves of the experimental equipment and model when the input signal frequency is 1 Hz.

Parameter identification based on the fractional-order Hammerstein rate-dependent nonlinear hysteresis model requires two steps to complete, that is:

1. Bouc-Wen model identification. This model mainly reflects the static nonlinear hysteresis characteristics of the PEA. The model parameters and identification results are shown in Table 2 and Figure 7.
2. Identification of the fractional-order model, which mainly reflects the linear dynamic characteristics of the PEA. A sine frequency sweep signal with a frequency range of 1–100 Hz is generated by Matlab as the input of the Bouc-Wen model. The output data of the Bouc-Wen model is used as the input of the fractional-order model, and the collected output data of the experimental equipment of the frequency sweep signal is used as the output of the fractional-order model. Through the input and output data of the aforementioned fractional model, the DE-ABC is used to identify the parameters of the fractional model. Through the DE-ABC, the fractional linear dynamic model is:
  (13) $$G\left(s\right)=\frac{5.2589\times 10^{6}}{s^{2.0384}+3081s^{1.0523}+5.4955\times 10^{6}}$$

### 4.4. Model Examination

In this section, the effectiveness of the rate-dependent Hammerstein model is testified when the PEA receives input signals of different frequencies.

**Figure 8:**
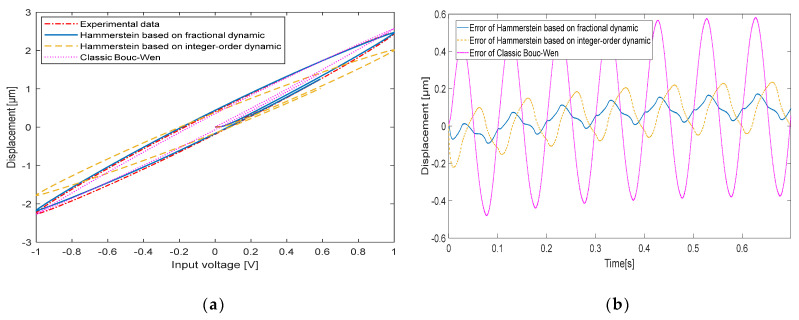
The comparison between experimental data and the Hammerstein model at 10 Hz: (**a**) Hysteresis loop; (**b**) Time curve.

**Figure 9:**
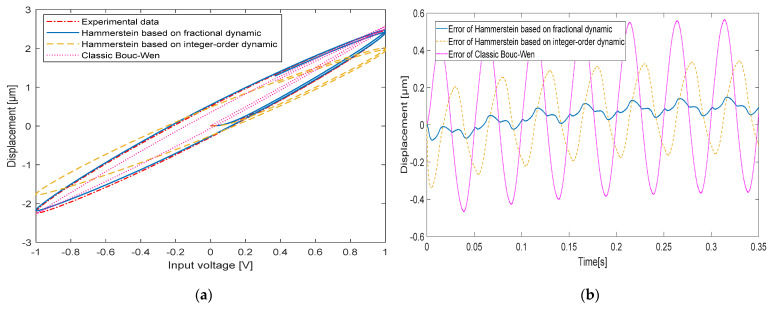
The comparison between experimental data and the Hammerstein model at 20 Hz: (**a**) Hysteresis loop; (**b**) Time curve.

**Figure 10:**
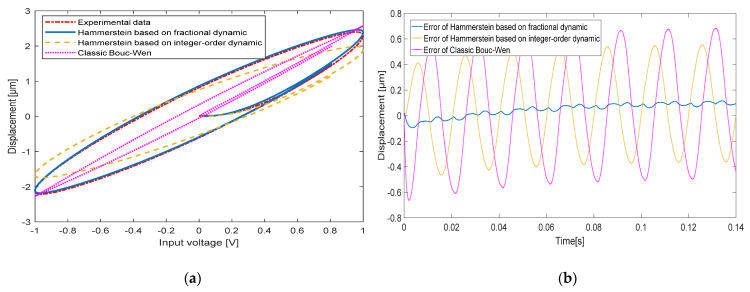
The comparison between experimental data and the Hammerstein model at 50 Hz: (**a**) Hysteresis loop; (**b**) Time curve.

**Figure 11:**
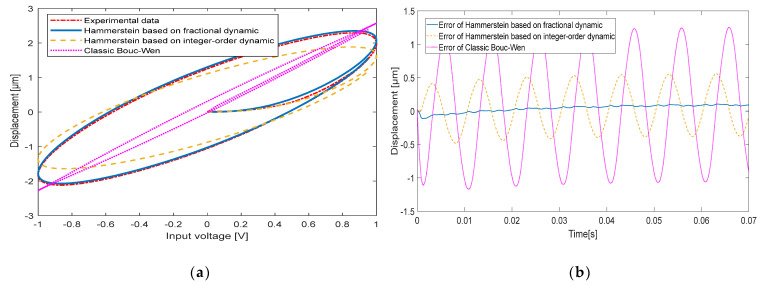
The comparison between experimental data and the Hammerstein model at 100 Hz: (**a**) Hysteresis loop; (**b**) Time curve.

When the input signal frequencies are 10 Hz, 20 Hz, 50 Hz and 100 Hz, respectively, the comparison between the experimentally measured hysteresis shape and the hysteresis shape simulated by the classical Bouc-Wen model, the hysteresis shape simulated by the Hammerstein model based on the integer-order dynamic model and the hysteresis shape simulated by the Hammerstein model based on the fractional-order dynamic model is shown in Figure 8, Figure 9, Figure 10 and Figure 11, respectively. Table 3 shows the hysteresis shape simulated by the classic Bouc-Wen model, the hysteresis shape simulated by the Hammerstein model based on the integer-order dynamic model, and the maximum error(Max error) and the mean square error(RMSE) of the Hammerstein model based on the fractional-order dynamic model.

When the input signal frequency of the piezoelectric actuator is 10 Hz, a Max error of the classic Bouc-Wen model is 0.5000 μm, and a RMSE is 0.1127. The Hammerstein model based on the integer-order dynamic model has a Max error of 0.1144 μm and a RMSE of 0.0413. The Hammerstein model based on the fractional dynamic model has a Max error of 0.0753 μm and a RMSE of 0.0264.

When the input signal frequency of the piezoelectric actuator is 20 Hz, a Max error of the classic Bouc-Wen model is 0.5161 μm and a RMSE is 0.1095. The Hammerstein model based on the integer-order dynamic model has a Max error of 0.2572 μm and a RMSE of 0.0627. The Hammerstein model based on the fractional dynamic model has a Max error of 0.0915 μm and a RMSE of 0.0244.

When the input signal frequency of the piezoelectric actuator is 50 Hz, a Max error of the classic Bouc-Wen model is 0.6292 μm and a RMSE is 0.1411. The Hammerstein model based on the integer-order dynamic model has a Max error of 0.5266 μm and a RMSE of 0.1086. The Hammerstein model based on the fractional dynamic model has a Max error of 0.0788 μm and a RMSE of 0.0228.

When the input signal frequency of the piezoelectric actuator is 100 Hz, a Max error of the classic Bouc-Wen model is 1.2012 μm and an RMSE is 0.2698. The Hammerstein model based on the integer-order dynamic model has a Max error of 0.5039 μm and a RMSE of 0.1101. The Hammerstein model based on the fractional dynamic model has a Max error of 0.0728 μm and an RMSE of 0.0221.

It can be known by analyzing the experimental results. The traditional Bouc-Wen model can only describe the symmetrical and rate-independent hysteresis characteristics. However, in actual conditions, the hysteresis characteristics of experimental equipment are often asymmetric and rate-dependent. It can be known from Table 3 that as the voltage frequency increases, the error of the traditional Bouc-Wen model begins to increase. The Hammerstein model based on the integer- order dynamic model can reveal the asymmetric and rate-dependent hysteresis characteristics. However, because the fractional-order model is more in line with the engineering practice and can highlight the dynamic characteristics of piezoelectric actuator more accurately, the Hammerstein model using the fractional order model to describe the dynamic characteristics of PEA is more accurate.

## 5. Conclusions

This paper proposes a rate-dependent hysteresis model based on fractional Hammerstein. The Bouc-Wen model describes the nonlinear static features of the piezoelectric actuator, and the fractional model describes the dynamic features of the piezoelectric actuator. First, the DE-ABC is used to identify the Bouc-Wen model parameters with static hysteresis. Secondly, on the basis of this Bouc-Wen static hysteresis model, the fractional-order dynamics model is obtained through the input and output data of the sweep signal with a frequency of 1–100 Hz. The DE-ABC is applied to the parameter identification of the Bouc-Wen model and fractional order model. The simulation results show that in the wide frequency range of 1–100 Hz, the Max error is about 0.0915 μm, and the RMSE is 0.0244. In summary, when the excitation voltage frequency of the piezoelectric actuator is at different frequencies, the Hammerstein model based on the fractional-order proposed in this paper can provide higher accuracy at different frequencies.

## Figures and Tables

**Table 1:** Best fitness of each algorithm.

Algorithms	Best Fitness Value
DE	0.0247
ABC	0.0144
DE-ABC	0.0120

**Table 2:** The parameters of the model.

x	y	α	β	γ	RE	RMSE
0.0031	2.0000	−1.2039	0.1111	0.0024	0.0208	0.0120

**Table 3:** RMSE and Max error of the model.

Frequency	Model	Max Error	RMSE
10 Hz	Classic Bouc-Wen	0.5000 μm	0.1127
	Hammerstein based on integer-order dynamic	0.1144 μm	0.0413
	Hammerstein based on fractional dynamic	0.0753 μm	0.0264
20 Hz	Classic Bouc-Wen	0.5161 μm	0.1095
	Hammerstein based on integer-order dynamic	0.2572 μm	0.0627
	Hammerstein based on fractional dynamic	0.0915 μm	0.0244
50 Hz	Classic Bouc-Wen	0.6292 μm	0.1411
	Hammerstein based on integer-order dynamic	0.5266 μm	0.1086
	Hammerstein based on fractional dynamic	0.0788 μm	0.0228
100 Hz	Classic Bouc-Wen	1.2012 μm	0.2698
	Hammerstein based on integer-order dynamic	0.5039 μm	0.1101
	Hammerstein based on fractional dynamic	0.0727 μm	0.0221
